# Mechanistic and Structural Understanding of Uncompetitive Inhibitors of Caspase-6

**DOI:** 10.1371/journal.pone.0050864

**Published:** 2012-12-05

**Authors:** Christopher E. Heise, Jeremy Murray, Katherine E. Augustyn, Brandon Bravo, Preeti Chugha, Frederick Cohen, Anthony M. Giannetti, Paul Gibbons, Rami N. Hannoush, Brian R. Hearn, Priyadarshini Jaishankar, Cuong Q. Ly, Kinjalkumar Shah, Karen Stanger, Micah Steffek, Yinyan Tang, Xianrui Zhao, Joseph W. Lewcock, Adam R. Renslo, John Flygare, Michelle R. Arkin

**Affiliations:** 1 Department of Biochemical and Cellular Pharmacology, Genentech, Inc., South San Francisco, California, United States of America; 2 Department of Structural Biology, Genentech, Inc., South San Francisco, California, United States of America; 3 Small Molecule Discovery Center, University of California San Francisco, San Francisco, California, United States of America; 4 Department of Discovery Chemistry, Genentech, Inc., South San Francisco, California, United States of America; 5 Department of Early Discovery Biochemistry, Genentech, Inc., South San Francisco, California, United States of America; 6 Department of Neuroscience, Genentech, Inc., South San Francisco, California, United States of America; Universite de Sherbrooke, Canada

## Abstract

Inhibition of caspase-6 is a potential therapeutic strategy for some neurodegenerative diseases, but it has been difficult to develop selective inhibitors against caspases. We report the discovery and characterization of a potent inhibitor of caspase-6 that acts by an uncompetitive binding mode that is an unprecedented mechanism of inhibition against this target class. Biochemical assays demonstrate that, while exquisitely selective for caspase-6 over caspase-3 and -7, the compound’s inhibitory activity is also dependent on the amino acid sequence and P1’ character of the peptide substrate. The crystal structure of the ternary complex of caspase-6, substrate-mimetic and an 11 nM inhibitor reveals the molecular basis of inhibition. The general strategy to develop uncompetitive inhibitors together with the unique mechanism described herein provides a rationale for engineering caspase selectivity.

## Introduction

Caspases are a family of cysteinyl proteases that are key mediators of apoptosis and inflammation [1], [2]. The apoptotic “executioner” caspases (caspases-3, -6 and -7) are translated as proenzymes containing a short pro-domain, a p20 subunit, a linker region, and p10 subunit. Their canonical activation mechanism involves proteolysis by “initiator” caspases (caspases-8 and -9) at three distinct sites to remove the prodomain and linker region [3]–[6]. The resulting active enzyme is a dimer, wherein each subunit contains a p10 and p20 chain and one active site. The caspase enzymatic mechanism is similar to other cysteine proteases; substrate binds to the active site to form the Michaelis complex, a covalent tetrahedral intermediate is formed by attack of the active-site thiolate cysteine on the scissile carbonyl, the substrate amide bond is cleaved to generate an acyl enzyme intermediate, and the intermediate is hydrolyzed by water to yield the new substrate C-terminus and apo-enzyme [7]. Active caspases are capable of cleaving numerous cellular proteins [8], [9] and carrying out the terminal phase of cell death signaling.

Due to the role of caspase-6 in neurodegeneration [10]–[14], there is strong interest in developing selective, small-molecule inhibitors of this enzyme. This family of proteases has proven resistant to traditional methods of drug discovery, however, and most known inhibitors contain a covalent warhead, significant peptidic character, and/or an aspartic acid. Each of these characteristics reduces the potential for caspase selectivity, cell permeability, and blood-brain barrier penetrance. For instance, the traditional caspase probes used in biological assays are tetrapeptides containing the ideal substrate sequences for each caspase and a covalent warhead that reversibly or irreversibly modifies the active-site cysteine. These tools lack the necessary caspase selectivity profiles to facilitate the delineation of isoform-specific signaling pathways in a cellular context [15]. To address these challenges, a number of alternative chemical approaches have been used. Leyva, et al, recently disclosed the design of novel, nonpeptidic inhibitors identified through “substrate assisted screening”; while potent, these compounds are non-selective and still contain an irreversible covalent warhead [16]. There has also been significant interest in developing noncompetitive or allosteric inhibitors, with the idea that non-active site binding could achieve greater selectivity and improved physicochemical properties over competitive inhibitors [17], [18]. This notion is supported by the discovery of an allosteric site at the dimer interface of caspases 1, 3, and 7. Applying the disulfide-trapping (Tethering) method of fragment discovery, scientists at Sunesis Pharmaceuticals identified fragments that bound at the dimer interface and inhibited enzymatic activity [19], [20]. These fragments were not tested for cellular activity, and the druggability of this site remains an interesting, open question.

Using a fluorogenic assay platform we identified a series of molecules that inhibit caspase-6 in an unexpected and mechanistically uncompetitive fashion. Detailed structural and mechanistic studies with the most potent of these compounds indicate that it binds to the enzyme-substrate complex in a highly specific manner to inhibit substrate turnover. This uncompetitive mechanism of enzyme inhibition is novel for any of the caspase family members. The present compound demonstrates a very distinctive molecular recognition for caspase-6/VEID peptides, and points the way towards utilizing uncompetitive inhibition as a strategy for the discovery of highly selective caspase inhibitors.

## Experimental Procedures

### Expression and Purification of Caspase-6

Cloning, expression, and purification of caspase-6 for enzymatic assays is described in Experimental Procedures S1.

### Caspase Enzymatic Assays

The in vitro enzymatic caspase assays utilize synthetic tetrapeptide substrates labeled with the fluorophores Rhodamine110 (R110) or 7-amino-4-methylcoumarin (AMC) at the P1 aspartic acid (Asp) residue. All assays were performed in 384-well plates in 12 µL reaction volume consisting of enzyme, substrate and indicated concentration of inhibitor or DMSO in assay buffer (50 mM HEPES [pH 7.0], 25 mM MgSO4, 0.5 mM EGTA, 5 mM Glutathione (GSH), 0.01% Triton X-100 containing 0.1% Bovine Gamma Globulin (BGG)). All inhibitors were serially diluted in 100% DMSO prior to dilution in assay buffer and transfer to assay plate. DMSO was diluted into assay buffer similarly for blank wells (no enzyme or compound) and final DMSO concentration was 1%. The concentration of caspase-6 used in enzymatic reactions typically varied between 1–10 nM depending on substrate used. Unless otherwise indicated, substrate concentration was within 3-fold of the determined Km_apparent_ (5 µM (Ac-VEID)_2_R110 [Km_app_ = 8 µM]; 5 µM (Ac-DEVD)_2_R110 [Km_app_ = 8 µM]; 25 µM (Ac-IETD)_2_R110 [Km_app_ = 70 µM]; 25 µM (Ac-WEHD)_2_R110 [Km_app_ = 70 µM]; 10µM Ac-VEID-AMC [Km_app_ = 16µM]; 5 µM Ac-VEID-R110 [Km_app_ = 8 µM]). The concentration of substrate utilized in selectivity assays for each caspase isoform was also held as close to Km_apparent_ as reasonably achievable (Table S1). Caspases-3 and -7 were expressed and purified at Genentech as the catalytic domain consisting of large p20 and small p10 subunits without prodomain. For all caspase enzymatic assays, the reaction plate was incubated at room temperature for 40 minutes and then read on Envision (Perkin Elmer) fluorescent plate reader at excitation/emission wavelengths of 485/535 nm (R110) or 350/450 nm (AMC).

The caspase-6 HTS assay was conducted essentially as described above with following exceptions: assay buffer contained 20 mM Pipes [pH 7.2], 100 mM NaCl, 1 mM EDTA, 10% Sucrose, 0.1% Chaps, 10 mM Dithiothreitol (DTT); incubation time was 10 minutes; 10 µM (VEID)_2_R110 substrate was N-terminally capped with a benzyloxy (Z) group in lieu of an acetyl (Ac); fluorescence was monitored using an Analyst HT plate reader (Molecular Devices).

The assay to monitor cleavage of Lamin A by purified human caspase-6 is described in Experimental Procedures S1.

### Data Analysis

The endpoint fluorescent emission (RFU) in each well was plotted as a function of inhibitor concentration and the 50% inhibition (IC_50_) values were determined using a nonlinear least squares fit of the data to a four parameter equation using Prism 5.0 software (GraphPad Software, San Diego, CA). Ki values for VEID-CHO were calculated using this equation: Ki = IC_50_/([S]/Km +1). Ki values for Compound **3** were calculated using this equation: Ki = IC_50_/(Km/[S] +1). Concentration-response curves for each inhibitor were normalized to zero and 100% based on no enzyme or DMSO control, respectively. For steady-state kinetic analysis, initial reaction velocity (RFU/minute) was plotted against substrate concentration at each inhibitor concentration and the data was fit to a hyperbolic Michaelis-Menten model using Prism 5.0 software. Km (µM) and Vmax (RFU/minute) were calculated by using this equation: v = Vmax • [S]/Km+[S] where v = initial reaction velocity at indicated substrate concentration (S). Vmax values were normalized to zero and 100% based on no enzyme or DMSO control, respectively.

#### Chemical syntheses

The synthesis of uncompetitive caspase-6 inhibitors is described in Experimental Procedures S1.

#### Crystallization

Crystals of a binary enzyme-substrate (zVEID) complex were first generated by reacting active caspase-6 with a 1.5 molar excess of a benzyloxycarbonyl-VEID (zVEID) substrate possessing a 2,3,5,6-tetrafluorophenoxy leaving group for 4 hours. The reaction mixture was desalted and then concentrated to 6.5 mg/mL and crystallized in 12% (w/v) PEG3350, 0.2 M NaMalonate pH 4.0. Crystals of the binary complex of caspase-6/VEID were then soaked overnight with 1 mM of **3.**

#### X-ray data collection, structure determination and refinement

Diffraction data to 2.0Å resolution was collected at Advanced Photon Source beamline 21-ID-G (Table S4). The data was indexed, integrated and scaled using HKL2000 [21] the structure was solved by molecular replacement using the Casp6-zVEID structure as the search model (PDB-ID 3OD5). The initial FoFc electron density maps clearly show unambiguous density for **3** bound close to the VEID peptide in both active sites (PDB-ID 4HVA). The compound was fit to the density and the model was subjected to iterative cycles of refinement and rebuilding using Phenix and Coot [22], [23] (Table S4).

### Surface Plasmon Resonance

For SPR experiments, caspase-6 was cloned to include a C-terminal avi-tag (Avidity) and expressed and purified as above, except that biotin ligase (BirA) was co-expressed during fermentation. This resulted in an active caspase-6 protein with a single biotin molecule attached to the lysine in the avi-tag sequence. Avi-tagged zymogen C163A-caspase-6 was processed to mature C163A-caspase-6 by the addition of active caspase-3 and caspase-6. Chip preparation for neutravidin-based capture was performed as previously described using either a Biacore T100 or Biacore 3000 instrument (GE Healthcare) [24]. Running buffer was 50 mM HEPES pH 7.2, 100 mM MgSO_4_, 30 mM NaCl, 1 mM TCEP, 0.01% Triton X-100, 1% PEG-3350, 2.5% DMSO, and the instrument was set for 20 degrees C. After capture one flow cell of apo-caspase-6 was exposed to a continuous flow of 20 µM VEID-FMK. A rise in signal could be detected for the binding/reacting of the VEID-FMK and exposure was continued until no additional rise in response was observed (∼45 minutes) indicating full saturation of all binding sites. There was no observed decrease in signal upon washing, indicating the reaction was irreversible. Data were reduced, solvent correct, double referenced, and fit using the Scrubber II software package (BioLogic Software, Campbell, Australia; http://www.biologic.com.au). Estimation of the K_D_ for **3** binding to apo-caspase6 was done by locking the R_max_ of **3** to a higher-affinity, saturable, control compound as previously described [24]. Fluorescent substrates were too limiting in solubility and quantity to be added to the running buffer, so substrates were mixed at a concentration equal to their Km_app_ with **3** and injected together over the indicated surfaces.

### Molecular Modeling

Modeling of **3** bound to the Michaelis complex and to the acyl-enzyme intermediate formed by VEID-R110/caspase-6 is described in Experimental Procedures S1.

## Results

### Chemical Optimization of Screening Hits Yields Low Nanomolar Inhibitors

**Figure 1:**
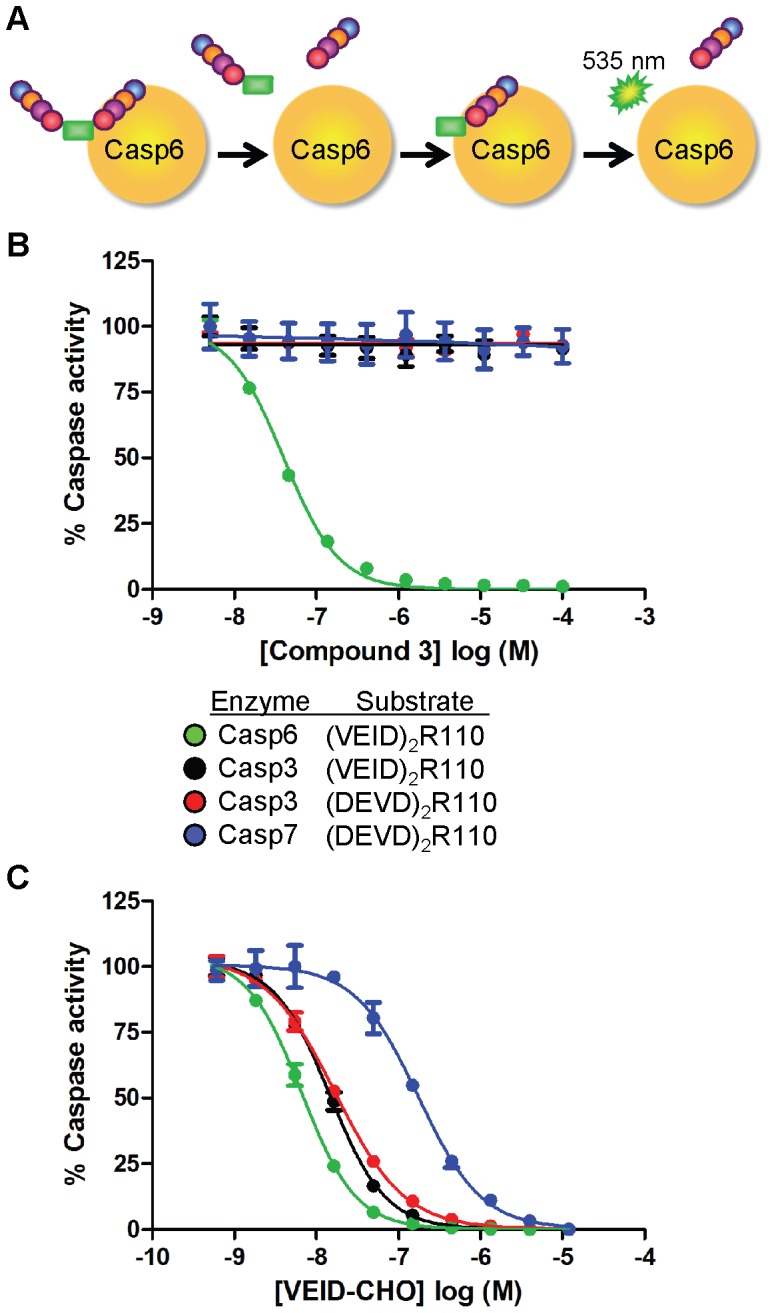
Inhibitor potency and selectivity against caspase family members. (A) Schematic of divalent tetrapeptide substrate proteolysis to release R110 fluorophore. Removal of both tetrapeptides by caspases is required for signal generation at 535 nm. Concentration-response analysis of compound **3** (B) and VEID-CHO (C) against caspase-6 (green), caspase-3 (black or red) or caspase-7 (blue). The particular divalent R110 peptide substrate used with each enzyme is indicated in the figure key and assay specifics can be found in Experimental Procedures. Potency values for (B–C) can be found in Table S2. Concentration response curves were generated in duplicate and represent 1 of at least 2 experiments with similar results. Each curve is normalized to zero and 100% based on no enzyme or DMSO, respectively. Data represent mean ± standard error of the mean.

**Figure 2:**
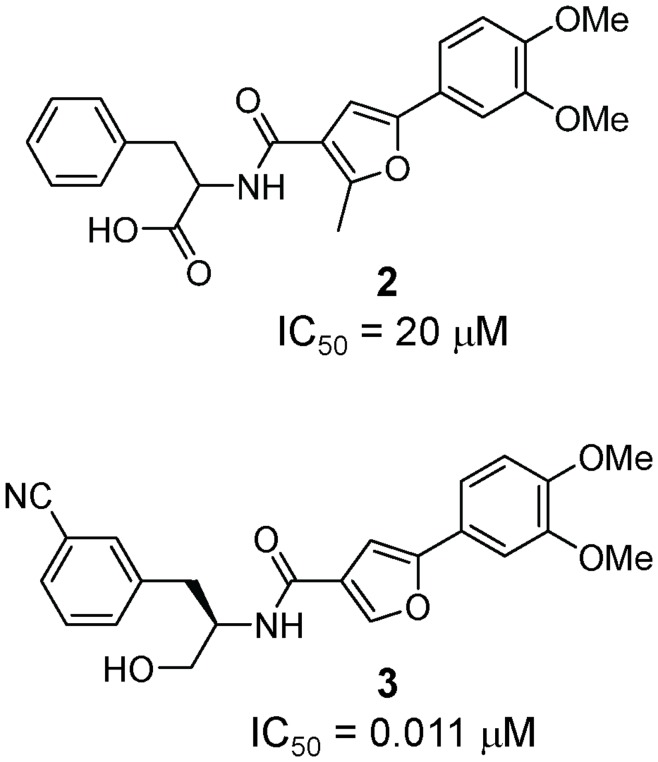
Structure of the *N*-furoyl-phenylalanine screening hit (2) and the optimized analog 3. Potency values represent the inhibition of caspase-6 cleavage of (VEID)_2_R110 substrate.

We developed and ran a screening assay that monitored inhibition of caspase-6 using a caged fluorophore substrate (Figure 1A). The substrate contained a Rhodamine110 (R110) dye conjugated to two valine-glutamate-isoleucine-aspartate (VEID) tetrapeptides; cleavage of both peptides from the dye yields maximal fluorescence. The original *N*-furoyl-phenylalanine screening hit (compound **2**) had undetermined stereochemical configuration and exhibited modest inhibition of caspase-6 (IC_50_ = 20 µM). Synthesis of authentic samples of both *R* and *S* enantiomers revealed that the *R* enantiomer, derived from the unnatural D-phenylalanine, was approximately 100-fold more potent than the *S* enantiomer. Based on potency and physicochemical properties, we selected compound **2** as a starting point for chemistry (manuscript in preparation). From this effort, we identified compound **3** with a potency of 11 nM (Figure 2). Compound **3** contains four changes that led to improved potency – use of the D-enantiomer at the amino acid, reduction of the acid to an alcohol, removal of the methyl group from the central furan ring, and addition of a meta-cyano substituent on the phenylalanine ring. Impressively, potency was increased 1,000-fold relative to the original hit **2** without an increase in molecular weight, resulting in a gain in the binding efficiency index (BEI; defined as pIC_50_/molecular weight) [25] from 11.5 to 19.7).

### Compound 3 Selectively Inhibits Caspase-6

To determine whether compound **3** was selective for caspase-6 relative to the other executioner caspases, we monitored the activity of caspases-3 and -7 using divalent tetrapeptide R110 substrates containing the DEVD consensus cleavage site. Compound **3** possesses near absolute selectivity for inhibition of caspase-6 cleavage of (VEID)_2_R110 compared to the other caspase family members tested (Figure 1B; Table S2). Similar selectivity profiles were observed for all compounds from this series tested in this manner. By contrast, a peptidic caspase inhibitor with aldehyde functionality (VEID-CHO) shows <35-fold selectivity across the three caspases (Figure 1C; Table S2).

### Compounds Possess Uncompetitive Mechanism of Inhibition

**Figure 3:**
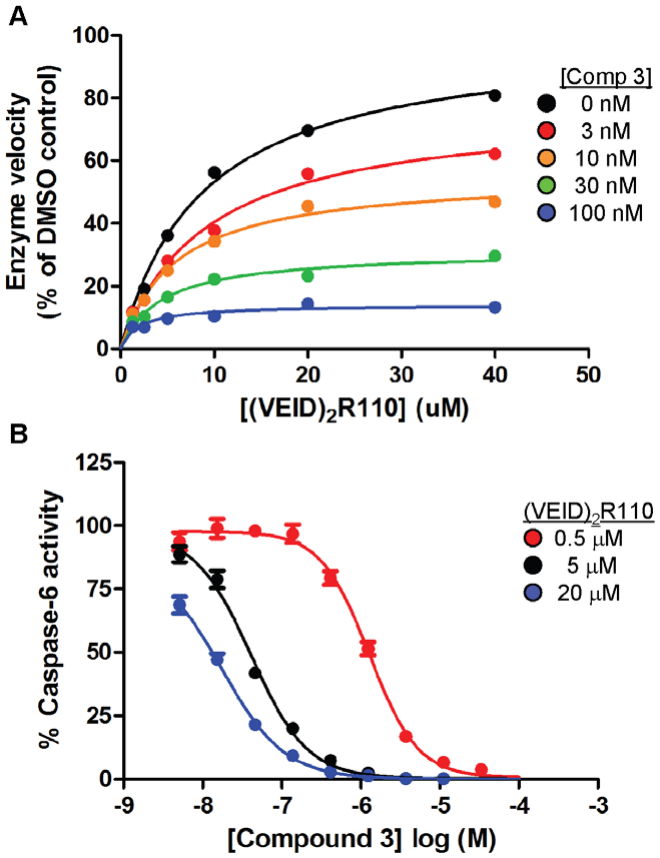
Kinetic caspase-6 enzymatic studies with compound 3 show uncompetitive mechanism of inhibition with (VEID)_2_R110 substrate. (A) The initial enzyme velocity of caspase-6 was plotted against the indicated concentration of (VEID)_2_R110 substrate in the presence of 0 nM (DMSO-black), 3 nM (red), 10 nM (orange), 30 nM (green) or 100 nM (blue) compound **3**. Double reciprocal plot of this data can be found in Figure S1 and Michaelis-Menten constants can be found in Table S3. (B) Concentration-response analysis of compound **3** when tested in the presence of 0.5 µM (red), 5 µM (black) or 20 µM (blue) (VEID)_2_R110 substrate. Michaelis-Menten kinetic experiments were performed with single points while concentration-response curves were performed in duplicate. Each data set represents 1 of at least 3 experiments with similar results.

We performed kinetic assays and determined the mechanism of inhibition (MOI) of compound **3**. As seen in Figure 3A and Figure S1, increasing concentrations of compound **3** resulted in decreasing Km values as well as a concomitant decrease in the Vmax (Table S3), indicative of an uncompetitive mechanism of inhibition. Thus, compound **3** binds to, and inhibits, the enzyme-substrate complex. The pharmacological significance of uncompetitive inhibition is that compound potency is enhanced as the substrate concentration in the reaction is increased (Figure 3B).

### Compound 3 Prefers VEID-based Peptide Substrates

**Figure 4:**
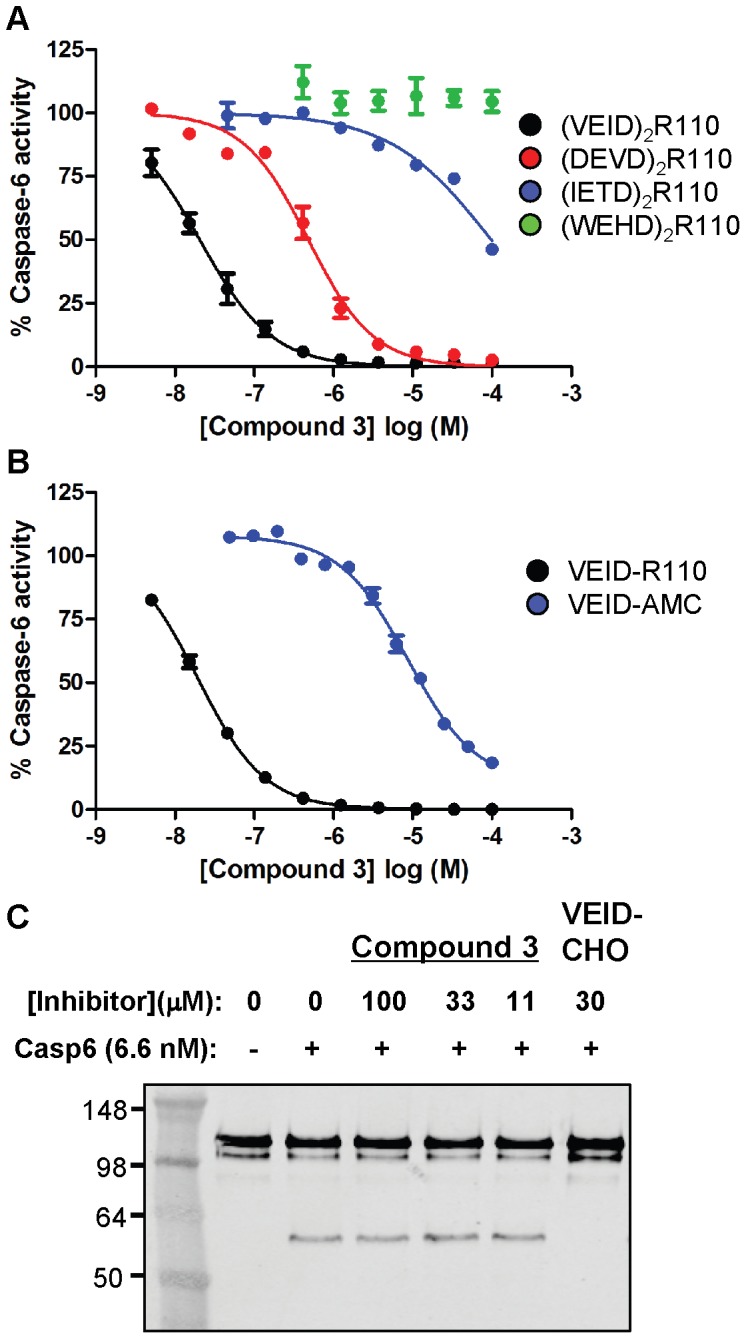
Compound 3 inhibition of caspase-6 is dependent on the substrate’s amino acid sequence and the P1’ character of the substrate. (A) Concentration-response analysis of compound **3** against caspase-6 cleavage of divalent R110-containing substrates with VEID (black), DEVD (red), IETD (blue) or WEHD (green) amino acid tetrapeptides. Each assay was performed using substrate concentrations within 3-fold of the Km_apparent_. (B) Concentration-response analysis of compound **3** against caspase-6 cleavage of monovalent VEID-based substrates with R110 (black) or AMC (blue) fluorophores conjugated to the C-terminal aspartate residue. (C) The indicated concentration of compound **3** or VEID-CHO was incubated with caspase-6 and GST-Lamin A prior to detection of cleaved Lamin A by western blotting. Only VEID-CHO was capable of inhibiting caspase-6 cleavage of recombinant Lamin A. Concentration response curves were generated in duplicate and represent 1 of at least 3 experiments with similar results. Each curve is normalized to zero and 100% based on no enzyme or DMSO, respectively. Western blot data represents 1 of at least 2 experiments.

Given the preferential binding of these inhibitors to a substrate/caspase-6 complex, we measured the inhibitory activity of **3** against a panel of related R110 substrates with alternative amino acid sequences. Because potency of uncompetitive inhibitors is dependent on the substrate concentration, care was taken for each assay to ensure substrate was included at concentrations approximating the measured Km_apparent_ (see Experimental Procedures). The inhibitory activity of **3** was very sensitive to the peptide substrate used to measure caspase-6 activity. For example, when caspase-6 activity was measured using (DEVD)_2_R110, the IC_50_ of compound **3** was 481 nM, ∼44-fold weaker than when monitored with (VEID)_2_R110 substrate (Figure 4A). Other substrates render **3** even less effective; (IETD)_2_R110 is inhibited only in the 100 µM range, where (WEHD)_2_R110 is not inhibited by **3** up to 100 µM. Similar shifts in potency upon transition from (VEID)_2_R110 to (DEVD)_2_R110 were observed with numerous other compounds from this series and is likely independent of Km disparity as both substrates possess near identical Km_apparent_ values. Further, the MOI of **3** as determined by Michaelis-Menten kinetics with (DEVD)_2_R110 substrate is also uncompetitive in nature (Figure S2A). While this compound inhibits caspase-6 cleavage of VEID or DEVD based substrates (albeit with varying potency), it is incapable of inhibiting caspase-3 cleavage of (VEID)_2_R110 (Figure 1B; Table S2). This suggests that the enzyme component in the enzyme/substrate complex confers a greater degree of selective binding than does the substrate component. In contrast, VEID-CHO equipotently inhibits caspase-3 cleavage of either substrate as would be expected for a competitive inhibitor (Figure 1C; Table S2).

To further investigate this unusual substrate-dependent behavior, we prepared monovalent VEID-R110 substrate, in which only one of the R110 amines is acylated with tetrapeptide. This substrate is inhibited by **3** as potently as the divalent (VEID)_2_R110, thus the second peptide plays no role in determining the potency of **3** (Figure 4B). On the other hand, the dye does play a strong role. VEID-AMC, in which the R110 is replaced by amino-methyl coumarin, is inhibited by **3** with an IC_50_ of 14 µM (∼750-fold loss in potency). Despite the marked loss in potency of this compound when AMC fluorophore is present in the substrate, the MOI as defined by Michaelis-Menten kinetics for these two monovalent substrates also supports an uncompetitive mechanism of inhibition (Figure S2B and unpublished results). In summary, inhibition of peptide/caspase-6 by these compounds is dependent on the sequence of the tetrapeptide on the N-side and the dye on the C-side (prime-side) of the scissile bond, but the MOI is consistently uncompetitive.

The sensitivity of compound **3** to different peptide substrates prompted us to explore caspase-6-dependent proteolysis of a biologically relevant full-length protein substrate containing the VEID cleavage motif. Lamin A is a nuclear envelope protein possessing two globular domains separated by a helical rod containing a VEID sequence known to be the site of caspase-6 proteolysis [26], [27]. Caspase-dependent digestion of recombinant Lamin A into two subunits is monitored via electrophoretic separation. As a positive control, Ac-VEID-CHO prevents 100% of cleavage at a concentration of 30 µM (Figure 4C). Compound **3** did not inhibit caspase-6 cleavage of recombinant Lamin A at 100 µM concentration.

### Structural Characterization of Compound 3 Bound to Z-VEID/caspase-6

**Figure 5:**
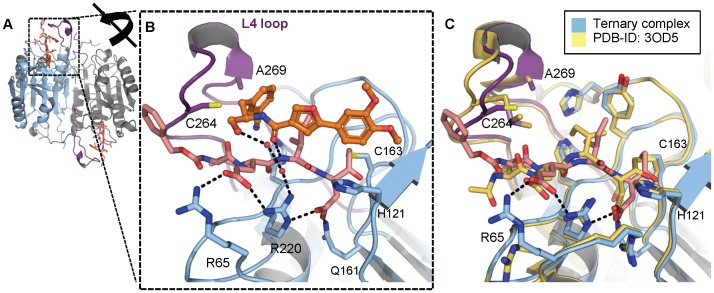
Crystal structure of caspase-6 ternary complex with 3 and covalently bound VEID inhibitor reveals the uncompetitive mechanism of this series of compounds. (A) Crystal structure of the ternary complex of caspase-6 with zVEID and compound **3** (PDB-ID 4HVA). The caspase-6 dimer is represented as cartoon with the A and B chains colored light blue and grey, respectively, and the L4 loop colored purple. The zVEID inhibitors are represented as sticks and are colored pink. Each inhibitor is covalently bound to the catalytic cysteine (Cys163) in both chain A and B. Two molecules of **3** are shown as ball and stick representation and colored orange. (B) Close up of the active site of chain A colored according to (A) with hydrogen bonds shown as black dashes. (C) Structural comparison of caspase-6 ternary complex with **3** bound (light blue) and caspase-6 binary complex with bound VEID-CHO (wheat) (PDB-ID 3OD5) illustrating the difference in the conformation of the tip of the L4 loop in the two crystal structures (residues 261–271).

In order to elucidate the molecular details of the uncompetitive MOI, we sought to determine the crystal structure of the ternary caspase-6/substrate/**3** complex. We first generated a binary complex of caspase-6 with a substrate surrogate covalently bound to the catalytic cysteine (Cys163) by incubating active caspase-6 with a covalent inhibitor (benzyloxycarbonyl (Z)-VEID-tetrafluorophenoxymethyl ketone). We observed that this inhibitor makes essentially the same interactions as previous reports of bound peptides with minor differences likely due to the additional methylene linker of this warhead compared to the aldehyde warhead used in other studies [6] (Figure 5).

Compound **3** was soaked into the crystal of the binary complex to yield a ternary complex of caspase-6/VEID/**3** (see Table S4 for x-ray statistics). The caspase-6/VEID portion of the ternary structure is very similar to the caspase-6/VEID binary complex (Figure 5C). The unambiguous electron density for **3** reveals a unique simultaneous binding of substrate and inhibitor that explains the uncompetitive behavior of this series (Figure 5A, 5B). The carbonyl group of **3** makes a 3.1-Å hydrogen bond with the backbone NH of the P2 Ile of the bound VEID substrate surrogate. The dimethoxyphenyl ring of **3** sits above the oxyanion hole created by the backbone NH group of Cys163; the 4-methoxy phenyl group displaces the water network around the His121-Cys163 catalytic dyad and the scissile bond. The furan ring does not make any specific interactions with the enzyme-substrate complex, and instead contributes to the active conformation of **3**. The primary alcohol of **3** makes a hydrogen bond interaction with the P3 Glu of VEID and participates in a water-mediated interaction with Arg220 of the L3 loop of caspase-6. The benzonitrile ring of **3** overlaps with the S4 subsite and tucks under the L4 loop of caspase-6, which places the nitrile group close to the sidechains of His168 from the L2 loop and His219 from the L3 loop. The crystal structure does not suggest a specific interaction between caspase-6 and the nitrile group even though the presence of the 3-CN is crucial for high potency inhibition (manuscript in preparation). The slight difference in the conformation of the L4 loop in the ternary complex in comparison to the conformation in the binary complex is likely due to the benzonitrile ring interaction with residues at the tip of the L4 loop (Figure 5). In summary, the x-ray structure of compound **3** supports the specificity observed by enzymology; the compound recognizes both the caspase-6 enzyme and the VEID substrate. The x-ray structure lacks the Rh110 dye, indicating that compound **3** can bind to the VEID/caspase-6 complex in the absence of a prime-side dye.

### Confirmation and Characterization of Ternary Complex Binding using Surface Plasmon Resonance (SPR)

Given that the affinity of compound **3** depends on the peptide sequence and presence of prime-side dye, an SPR-based assay was developed to characterize the binding affinity of **3** to catalytically dead (C163A mutation) as well as apo- and peptide inhibitor-bound forms of caspase-6. C163A-caspase-6 and Apo-caspase-6 were captured to different flow cells on a biosensor chip. One apo-caspase-6 surface was maintained in the apo-state while another was saturated with 20 µM Z-VEID-fluoromethyl ketone (Z-VEID-FMK) to produce the same binary Z-VEID/caspase-6 complex observed in X-ray crystallography.

**Figure 6:**
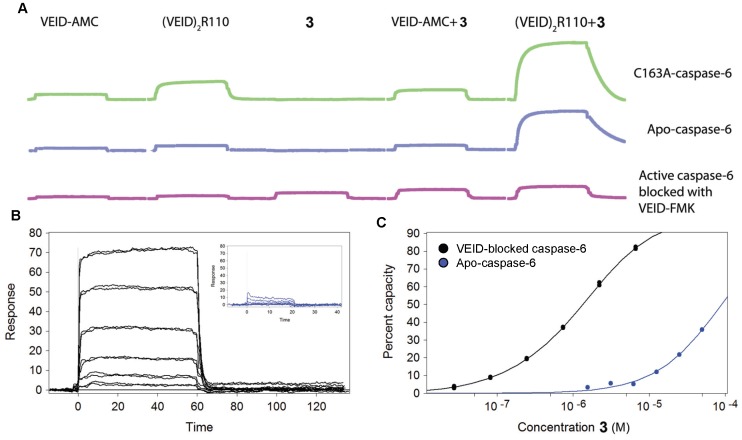
SPR detection of 3 binding to multiple caspase-6 surfaces confirms uncompetitive binding mode. (A) Catalytically inactive caspase-6 (green), apo-caspase-6 (blue) and caspase-6 saturated with VEID-FMK inhibitor (purple) were captured to chip surfaces and exposed to VEID-AMC, (VEID)_2_R110 and/or **3** to qualitatively monitor binding. Cooperative binding of **3** and (VEID)_2_R110 to C163 caspase-6 illustrate formation of the Michaelis-Menten complex. (B) Sensograms representing injections of escalating concentrations of **3** over VEID-FMK inhibitor-blocked caspase-6 surface (black). The inset represents similar injections of **3** over an unblocked apo-caspase-6 surface (blue). (C) Concentration-response analysis of data from (B) when compound **3** was injected over VEID-blocked caspase-6 surface (black) and apo-caspase-6 (blue) surfaces.

VEID-AMC (10 µM), (VEID)_2_R110 (10 µM) and **3** (1 µM) were injected alone or in combination over all three surfaces (Figure 6A). Minimal binding was observed with VEID-AMC across all proteins while more (VEID)_2_R110 bound to the C163A-caspase-6, consistent with substrate binding but inability of the catalytically dead caspase-6 to convert substrate to products. The greater degree in binding observed with (VEID)_2_R110 versus VEID-AMC to the C163A-caspase-6 surface is likely attributable to the larger molecular weight of the divalent substrate combined with the higher concentration of substrate relative to Km_app_. The binding of **3** was only detected to the VEID blocked surface and was not modulated by the addition of VEID-AMC or (VEID)_2_R110 substrates, as expected due to blockage of the peptide binding site by VEID-FMK. However, the apo-caspase-6 and C163A-caspase-6 surfaces show a dramatically larger response when co-injected with (VEID)_2_R110 and **3** compared to injection of **3** itself, directly confirming the uncompetitive-binding mode of the interaction. Qualitatively, the data indicate a significantly higher affinity of these two interactions than **3**+ (VEID)_2_R110 with VEID-blocked caspase-6. The clearly slower off-rate can be fit to generate an apparent K_D_ of ∼200 nM which represents the dissociation of both the compound and substrate. The same increase in response and apparent affinity improvement is not observed when **3** is co-injected with VEID-AMC, confirming the importance of the rhodamine-containing substrate for high-affinity binding and inhibition.

We observed very weak binding of **3** to apo-caspase-6 (K_D_ = 192 µM) while binding to the covalent VEID/caspase-6 complex demonstrated saturable 1∶1 binding and a two-log improvement in the K_D_ to 1.3 µM (Figure 6B and 6C). These observations are consistent with compound binding being uncompetitive with respect to the peptide substrate. The difference between this K_D_ and the enzymatic IC_50_ values (11 nM for VEID-R110, 14 µM for VEID-AMC) can be attributed to: 1) Use of fully VEID-saturated caspase-6 in the SPR experiments whereas the enzyme assays use cleavable substrates at a concentration equal to their Km_app_, 2) binding to the stable acyl-enzyme complex present in the SPR experiment versus the tetrahedral intermediate in the enzyme assays, and/or 3) occupation of the prime-side pocket with fluorophore in the enzyme assays. In any event, these data show that the presence of a P1’ fluorophore is not required for binding of compound **3** to VEID/caspase-6, but the presence and character of this fluorophore directly leads to additional compound-substrate interactions that modulate binding affinity.

## Discussion

**Figure 7:**
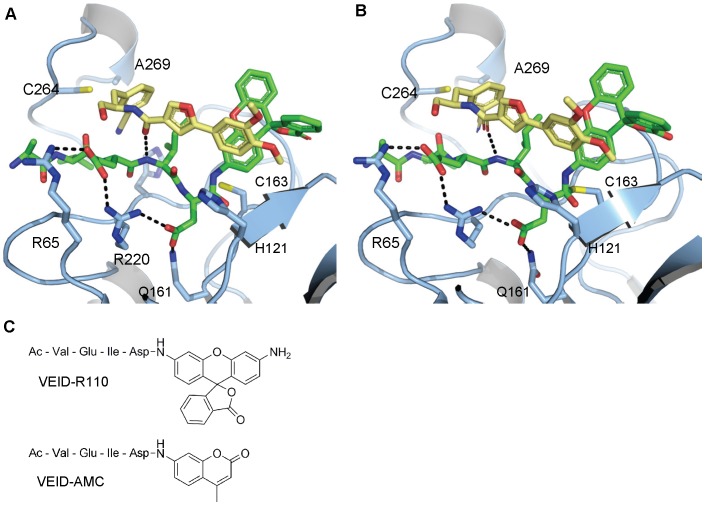
Docking models of caspase-6/VEID-R110/3 ternary complex explains fluorophore-dependent potency of this series of compounds. (A) Docking model of the Michaelis-Menten complex formed between caspase-6 (light blue), VEID-R110 (green sticks) and **3** (wheat sticks). (B) Docking model of the tetrahedral intermediate between caspase-6, VEID-R110 (green sticks) and **3** (wheat sticks) with substrate covalently bound to Cys163. (C) Depiction of monovalent VEID substrates with R110 or AMC fluorophores.

Our search for caspase-6 inhibitors led to the identification of a highly selective molecule that inhibits the enzyme via a novel mechanism not previously described for any of the caspases. Although it has recently been demonstrated for another cysteine protease that the acyl-enzyme intermediate is the primary resting state during the catalytic cycle [28], stabilization of this intermediate by **3** can be ruled out as the sole mechanism of inhibition, since no fluorophore dependence would be expected if this were the case. Therefore, there are two possible mechanisms by which these inhibitors may prevent cleavage of substrate: 1) stabilization of the Michaelis complex or 2) stabilization of the tetrahedral intermediate. To gain further structural insight into these possibilities we developed two models of the caspase-6/VEID-R110/**3** ternary complex, one with unbound substrate to represent the Michaelis complex and one with substrate covalently bound to illustrate the tetrahedral intermediate. First**,** a model for the covalently bound tetrahedral intermediate was constructed by the covalent docking of a truncated substrate model to the caspase-6/**3** complex followed by attachment of the R110 fluorophore (Figure 7B). This complex was then refined using Prime (Prime, version 2.2, Schrodinger, LLC, New York, NY, 2010) and MacroModel (MacroModel, version 9.8, Schrodinger, LLC, New York, NY, 2010). The Michaelis complex model was derived by breaking the cysteine-substrate bond in the covalent model and performing a constrained optimization of the complex where the inhibitor, substrate and catalytic dyad residues were permitted to move freely (Figure 7A) (details in Experimental Procedures S1). Both models provided low energy structures with plausible intermolecular contacts. Our existing data suggest that both mechanisms – binding to the ternary complex and to the tetrahedral intermediate – are important.

With respect to MOI scenario #1, we observe cooperative binding of **3** with (VEID)_2_R110 or VEID-AMC to catalytically-dead (Cys163Ala) caspase-6 by SPR (Figure 6A). This result indicates that the **3**/Michaelis complex can form, but it does not speak to whether **3** is able to prevent progress of the reaction, as would be required for inhibition. If **3** does indeed stabilize this complex to prevent formation of the tetrahedral intermediate, a possible mechanism is that **3** perturbs the oxyanion hole, inhibiting creation of the electrophilic carbonyl needed for attack. With respect to MOI scenario #2, our model also suggests that **3** could bind to the tetrahedral intermediate formed by addition of Cys163 to the amide bond (Figure 7B). We observe by x-ray crystallography that the dimethoxy phenyl ring of **3** disrupts the water network around the catalytic His121. Thus it is possible that if **3** prevents collapse of the tetrahedral intermediate, it could do so by perturbing the local environment around this key residue, preventing it from acting as the general acid. Although we are unable to isolate and quantify the binding interactions of **3** to the tetrahedral intermediate, it is noteworthy that the measured affinities of **3** to the Michaelis complex (∼200 nM by SPR) and acyl enzyme (1.3 µM by SPR) are both weaker than the potency determined in enzymatic assays (11 nM IC_50_). We speculate that binding of **3** to the tetrahedral intermediate is the favored enzyme/substrate complex leading to potent inhibition.

An unexpected feature of this inhibitor is the 2–3 orders of magnitude difference in inhibitory potency depending on the fluorophore employed in enzymatic assays, and the apparent lack of activity when fluorophore-free substrates are utilized. The computational models suggest one possible explanation for this difference, namely a polarized CH-π interaction between the para-methoxy group of **3** and the face of the orthogonal phenyl ring of the R110 dye, an interaction that is not possible with AMC-based substrates or substrates lacking a dye (e.g. native protein substrates) (Figure 7C). The importance of such CH-π interactions has been noted previously [29]. Furthermore, there appears to be either an edge-face or π-stack interaction between the phenyl ring of the inhibitor and the fluorophore aromatic ring. The remaining interaction energy difference can be explained by displacement of waters by the two extra rings of the R110, and/or additional hydrophobic interactions between the extra two rings of R110 and the protein. All of these interactions would be absent in a peptide substrate lacking a fluorophore at the P1’ position. It is known from studies on caspase-3 that prime side interactions can lead to a significant increase in inhibitory potency; for instance, the addition of a benzoxazole moiety on the prime side of the Ac-DEVD α-ketoaldehyde peptide inhibitor increases the potency ∼300-fold against caspase-3 [30]. As for the inability of **3** to inhibit Lamin A cleavage, the presence of substrate residues more distal to the scissile bond (P5–P8) may alter the general conformation of the inhibitor binding site to disrupt the key L2–L4 interactions observed in Figure 5B. The importance of P5 for caspase-2 substrate recognition and catalysis has been described [31] and we speculate that the inhibitor binding site defined here may be altered by similar enzyme-substrate interactions.

This class of inhibitors also shows sensitivity to the peptide sequence of the substrate, and unprecedented selectivity for caspase-6. To better understand this selectivity profile, we superposed the caspase-3/DEVD coordinates onto the caspase-6/VEID/**3** ternary structure (Figure S3). Three residues lining the binding site of **3** provide a structural rationale for the selectivity of these inhibitors (Cys264 and Ala269 in the L4 loop and His209 in the L3 loop); we believe that Ala269 is the primary driver of caspase selectivity (amino acids depicted in Figure 5C). Ala269 is Phe256 in caspase-3 and Phe282 in caspase-7. These larger residues would hinder compound binding by clashing with the benzyl side chain of all inhibitors from this series. Our models also explain the substrate peptide sequence sensitivity of these inhibitors. The smaller Val residue in the substrate (DEVD)_2_R110 would produce a weaker hydrophobic interaction between the substrate and the benzyl side chain, while the larger Trp and His residues in the substrate (WEHD)_2_R110 would prevent inhibitor binding by clashing with the inhibitor side chain.

The substrate-dependent variation in potency minimizes the utility of these inhibitors as tools to understand target biology. This finding may also suggest that peptide surrogates used in biochemical assays have potential to contribute to misleading SAR for other series of inhibitors. This phenomenon is not specific to caspase-6. A common assay system used to profile the activity of the histone deacetylase enzymes also incorporates a proximal fluorophore attached to the C-terminus of a tetrapeptide. The crystal structure of this Arg-His-Lys-Lys-Coumarin substrate with HDAC8 illustrates direct interactions of the fluorophore with amino acid residue side chains [32]. Several reports make claim that SIRT activation by Resveratrol is an artifact of this fluorogenic assay [33], [34], although follow up work confirms the original findings [35]. Thus, it is advised that a detailed mechanistic characterization of hits, as described here, be performed early in the triage stage of lead identification campaigns, particularly when inhibitors with unusual mechanisms are found.

In summary, the mechanistic and structural information described here explains the selective and substrate-specific inhibition of caspase-6 by a novel series of inhibitors. Uncompetitive inhibition is a proven strategy for other targets including MEK1/2 [36]–[38] and IMPDH [39], [40] but because these compounds recognize a specific substrate-enzyme complex, they do not potently inhibit cleavage of other more physiologically relevant substrates. These particular inhibitors provide new insight into caspase selectivity, a topic of significant importance in drug discovery. This mechanism of uncompetitive inhibition is unique for any caspase family member and suggests that the discovery of inhibitors of specific, biologically relevant, enzyme-substrate complexes may be achievable. The observed binding of **3** to the acyl-enzyme when no fluorophore occupies the prime side (Figure 5 and Figure 6) suggests that elaboration of this series could lead to biologically relevant caspase-6 inhibitors. The work described herein provides a template for identification of uncompetitive caspase inhibitors as well as effective triage strategies of lead matter with novel mechanisms.

## Supporting Information

Figure S1 **Double-reciprocal Lineweaver-Burke plot of compound 3 with (VEID)_2_R110 substrate showing uncompetitive MOI.** Initial reaction velocities from nonlinear Michaelis-Menten kinetic experiment shown in Figure 3A was transformed to linear analysis for visualization.
(TIF)

Figure S2 **Kinetic caspase-6 enzymatic studies with compound 3 show uncompetitive mechanism of inhibition with (DEVD)_2_R110 and VEID-AMC substrates.** (A) The initial enzyme velocity of caspase-6 was plotted against the indicated concentration of (DEVD)_2_R110 substrate in the presence of 0 nM (DMSO-black), 30 nM (red), 100 nM (orange), 300 nM (green), 1,000 nM (blue), 3,000 nM (purple) or 10,000 nM (pink) compound 3. (B) The initial enzyme velocity of caspase-6 was plotted against the indicated concentration of VEID-AMC substrate in the presence of 0 µM (DMSO-black), 1.6 µM (red), 3.1 µM (orange), 6.3 µM (green), 12 µM (blue), 25 µM (purple) or 50 µM (pink) compound 3. Experiments were performed with single points and represent 1 of at least 2 experiments with similar results. Enzyme velocity is normalized to zero and 100% based on no enzyme or DMSO, respectively.
(TIF)

Figure S3 **Structural comparison of the caspase-6/3 ternary complex reveals the structural basis of the exquisite caspase selectivity of this series of compounds.** Superposition of the caspase-3/DEVD binary complex (2DKO) (light grey) onto the structure of the caspase-6/VEID/3 ternary complex (light blue). The three residue differences that would reduce the affinity of 3 for caspase-3 are highlighted in violet and numbered. 1 = Ala in caspase-6 and Phe in caspase-3 and caspase-7; 2 = Cys in caspase-6 and Ser in caspase-3 and caspase-7; 3 = His caspase-6 and Trp in caspase-3 and caspase-7.
(TIFF)

Table S1 **Kinetic rate constants and enzymatic reaction conditions for Caspases-3, -6 and -7.**
(DOCX)

Table S2 **Potency of VEID-CHO and compound 3 against Caspase-3, -6 and -7 cleavage of divalent rhodamine substrates.**
(DOCX)

Table S3 **Michaelis-Menten constants for (VEID)_2_R110 with compound 3.**
(DOCX)

Table S4 **Data Collection and Refinement of Compound 3 Complex.**
(DOCX)

Experimental Procedures S1 **Supplemental Methods.**
(DOCX)
