# Supplementary figures and images for: Mechanistic and Structural Understanding of Uncompetitive Inhibitors of Caspase-6
Source: PLoS One. 2012 Dec 5;7(12):e50864. doi: 10.1371/journal.pone.0050864 (PMC3515450; doi:10.1371/journal.pone.0050864)

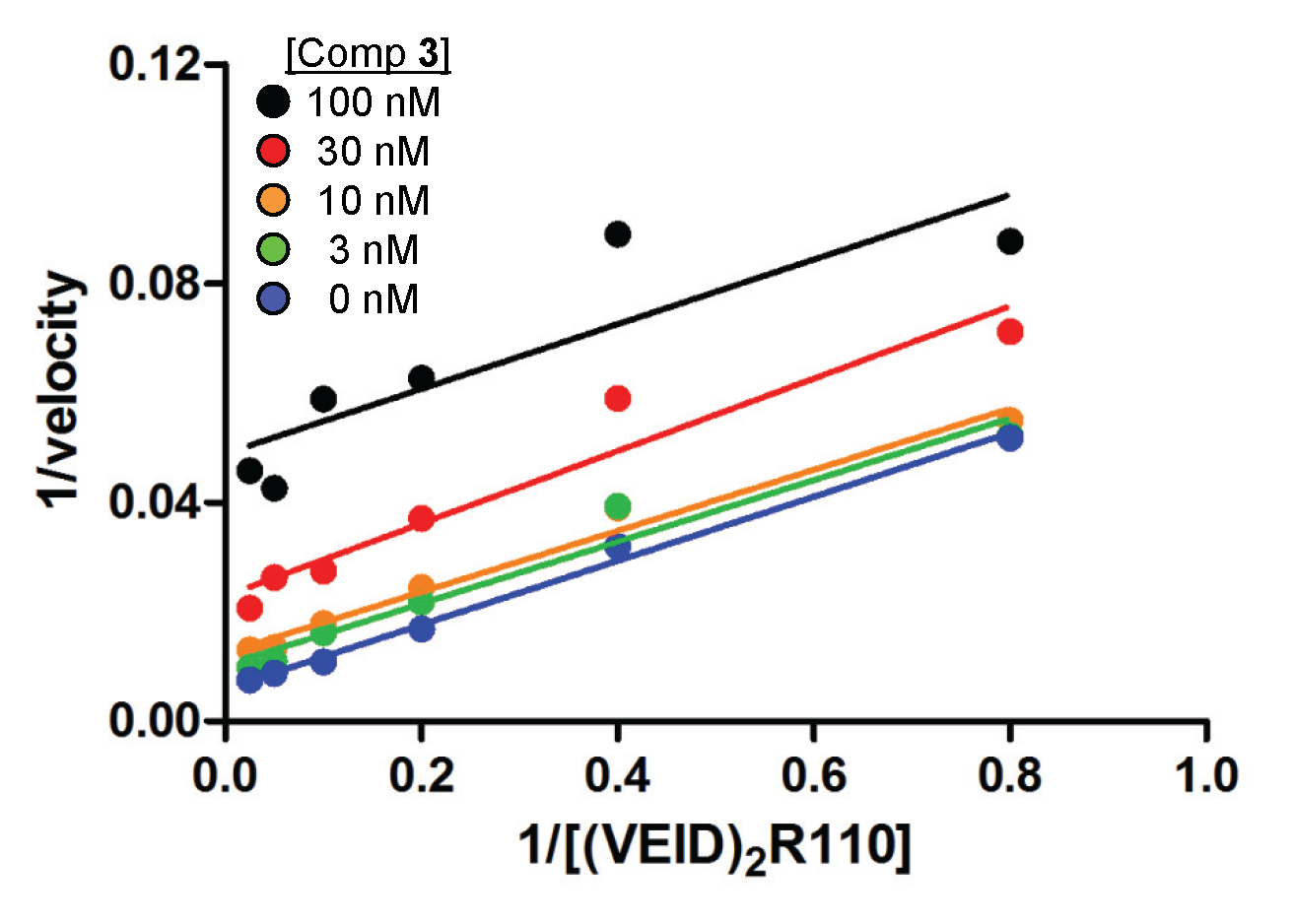

Supplement: Figure S1 — Double-reciprocal Lineweaver-Burke plot of compound 3 with (VEID)2R110 substrate showing uncompetitive MOI. Initial reaction velocities from nonlinear Michaelis-Menten kinetic experiment shown in Figure 3A was transformed to linear analysis for visualization. (TIF) [file pone.0050864.s001.tif]

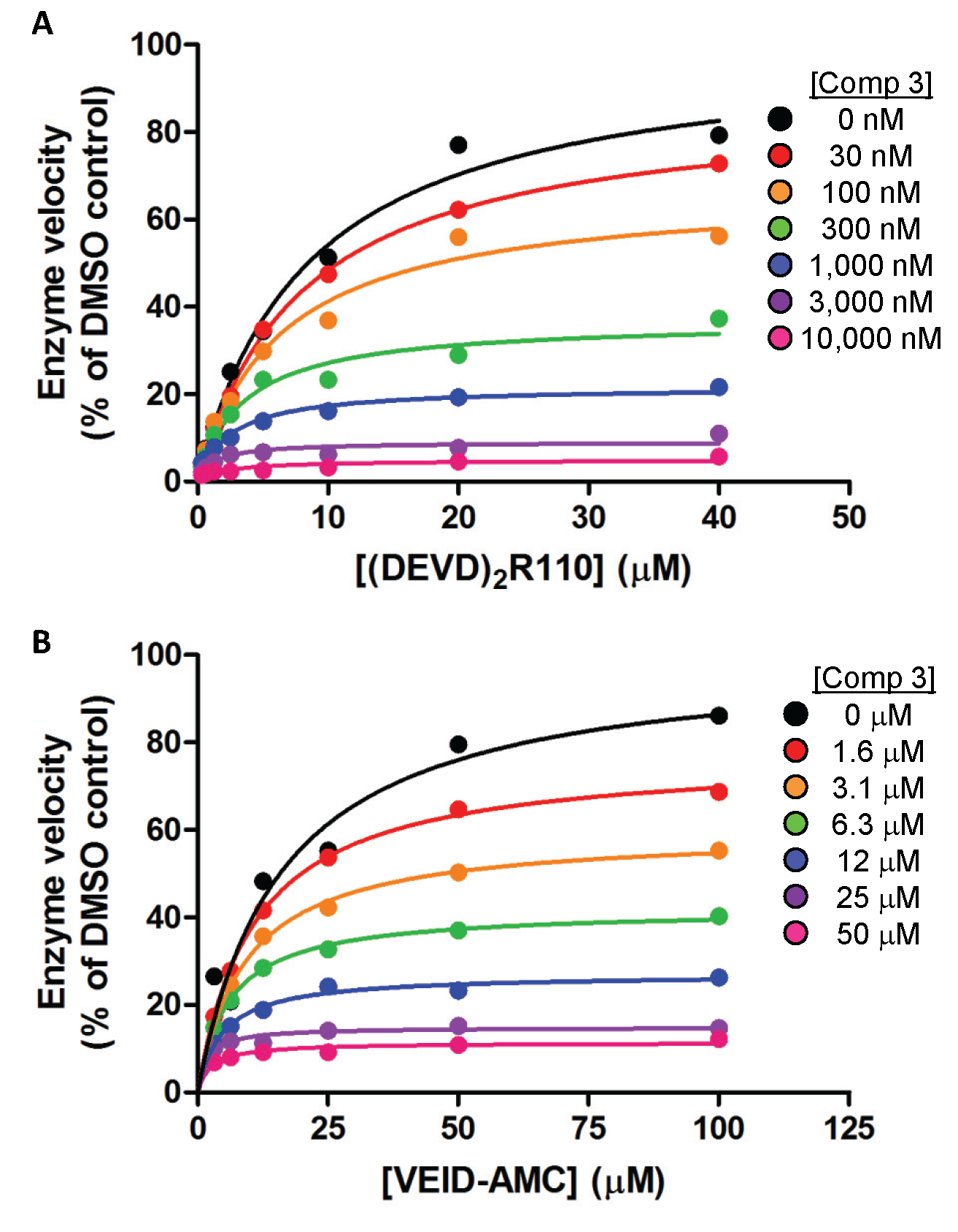

Supplement: Figure S2 — Kinetic caspase-6 enzymatic studies with compound 3 show uncompetitive mechanism of inhibition with (DEVD)2R110 and VEID-AMC substrates. (A) The initial enzyme velocity of caspase-6 was plotted against the indicated concentration of (DEVD)2R110 substrate in the presence of 0 nM (DMSO-black), 30 nM (red), 100 nM (orange), 300 nM (green), 1,000 nM (blue), 3,000 nM (purple) or 10,000 nM (pink) compound 3. (B) The initial enzyme velocity of caspase-6 was plotted against the indicated concentration of VEID-AMC substrate in the presence of 0 µM (DMSO-black), 1.6 µM (red), 3.1 µM (orange), 6.3 µM (green), 12 µM (blue), 25 µM (purple) or 50 µM (pink) compound 3. Experiments were performed with single points and represent 1 of at least 2 experiments with similar results. Enzyme velocity is normalized to zero and 100% based on no enzyme or DMSO, respectively. (TIF) [file pone.0050864.s002.tif]

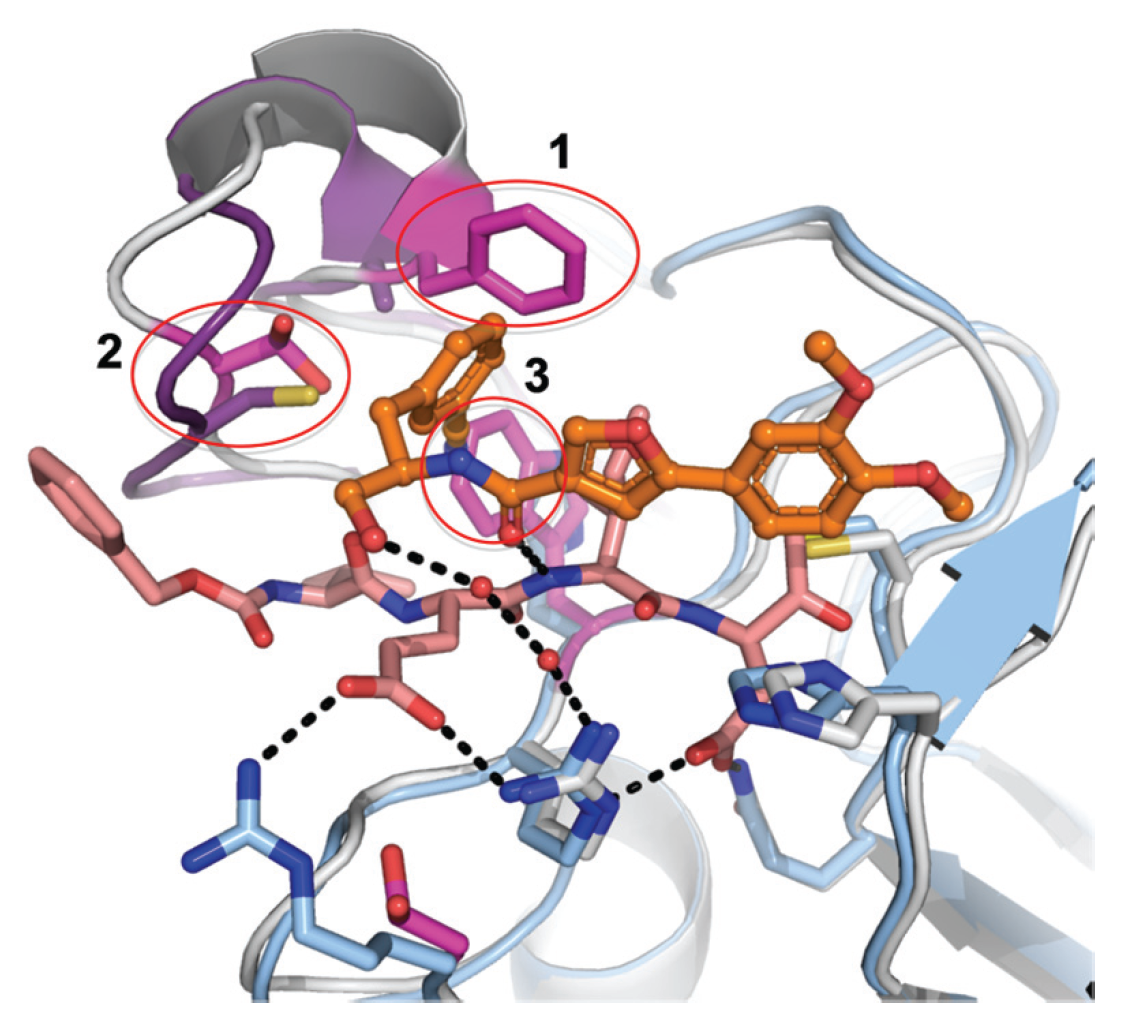

Supplement: Figure S3 — Structural comparison of the caspase-6/3 ternary complex reveals the structural basis of the exquisite caspase selectivity of this series of compounds. Superposition of the caspase-3/DEVD binary complex (2DKO) (light grey) onto the structure of the caspase-6/VEID/3 ternary complex (light blue). The three residue differences that would reduce the affinity of 3 for caspase-3 are highlighted in violet and numbered. 1 = Ala in caspase-6 and Phe in caspase-3 and caspase-7; 2 = Cys in caspase-6 and Ser in caspase-3 and caspase-7; 3 = His caspase-6 and Trp in caspase-3 and caspase-7. (TIFF) [file pone.0050864.s003.tiff]
