# Supplementary material for: Mechanistic and Structural Understanding of Uncompetitive Inhibitors of Caspase-6
Source: PLoS One. 2012 Dec 5;7(12):e50864. doi: 10.1371/journal.pone.0050864 (PMC3515450; doi:10.1371/journal.pone.0050864)
Supplement: Table S1 — Kinetic rate constants and enzymatic reaction conditions for Caspases-3, -6 and -7. (DOCX) [file pone.0050864.s004.docx]

| **Table S1**. Kinetic rate constants and enzymatic reaction conditions for Caspases-3, -6 and -7. | | | | | |
| --- | --- | --- | --- | --- | --- |
| Enzyme | Substrate | [Enzyme] (nM) | [Substrate] (µM) | K_cat_ (1/s) | Km_app_ (µM) |
| Caspase-6 | (VEID)_2_R110 | 3 | 5 | 0.11 | 8 |
| Caspase-6 | (DEVD)_2_R110 | 3 | 5 | 0.15 | 8 |
| Caspase-6 | (WEHD)_2_R110 | 10 | 25 | 0.004 | 70 |
| Caspase-6 | (IETD)_2_R110 | 10 | 25 | nd | 70 |
| Caspase-3 | (DEVD)_2_R110 | 0.03 | 1 | 9.6 | 0.9 |
| Caspase-3 | (VEID)_2_R110 | 1.3 | 5 | nd | 8 |
| Caspase-7 | (DEVD)_2_R110 | 0.005 | 1 | 58 | 2.8 |
| k_cat_ determinations were made using the following equation k_cat_ = Vmax/[E] and assume 100% active protein for each caspase.  nd = not determined | | | | | |
