# Supplementary material for: Mechanistic and Structural Understanding of Uncompetitive Inhibitors of Caspase-6
Source: PLoS One. 2012 Dec 5;7(12):e50864. doi: 10.1371/journal.pone.0050864 (PMC3515450; doi:10.1371/journal.pone.0050864)
Supplement: Table S2 — Potency of VEID-CHO and compound 3 against Caspase-3, -6 and -7 cleavage of divalent rhodamine substrates. (DOCX) [file pone.0050864.s005.docx]

| **Table S2**. Potency of VEID-CHO and compound **3** against Caspase-3, -6 and -7 cleavage of divalent rhodamine substrates. | | | |
| --- | --- | --- | --- |
|  |  | Mean IC_50_ / (Ki) (µM) | |
| Enzyme | Substrate | VEID-CHO | **3** |
| Caspase-6 | (VEID)_2_R110 | 0.007 / (0.004) | 0.011 / (0.004) |
| Caspase-3 | (DEVD)_2_R110 | 0.015 / (0.007) | >100 |
| Caspase-3 | (VEID)_2_R110 | 0.015 / (0.009) | >100 |
| Caspase-7 | (DEVD)_2_R110 | 0.175 / (0.129) | >100 |
