# Supplementary material for: Mechanistic and Structural Understanding of Uncompetitive Inhibitors of Caspase-6
Source: PLoS One. 2012 Dec 5;7(12):e50864. doi: 10.1371/journal.pone.0050864 (PMC3515450; doi:10.1371/journal.pone.0050864)
Supplement: Table S3 — Michaelis-Menten constants for (VEID)2R110 with compound 3. (DOCX) [file pone.0050864.s006.docx]

| **Table S3**. Michaelis-Menten constants for (VEID)_2_R110 with compound **3**. | | |
| --- | --- | --- |
|  | Constants for (VEID)_2_R110 | |
| Concentration (nM) of **3** | Km_(apparent)_ (µM) | Vmax (% of DMSO control) |
| 100 | 2.02 | 14.10 |
| 30 | 4.41 | 31.09 |
| 10 | 5.85 | 55.35 |
| 3 | 9.19 | 77.51 |
| 0 | 8.79 | 100 |
