# Supplementary material for: Mechanistic and Structural Understanding of Uncompetitive Inhibitors of Caspase-6
Source: PLoS One. 2012 Dec 5;7(12):e50864. doi: 10.1371/journal.pone.0050864 (PMC3515450; doi:10.1371/journal.pone.0050864)
Supplement: Table S4 — Data Collection and Refinement of Compound 3 Complex. (DOCX) [file pone.0050864.s007.docx]

**Table S4.** Data Collection and Refinement of Compound **3** Complex.

***Data collection*** APS 21-ID-G

space group P2_1_

unit cell (Å,˚) ***a***= 56 , ***b***= 62.6 , ***c***= 76.3 γ= 104.8

Resolution (Å) 30 – 2.0 ( – )

Rsyma,b 0.085 (0.358)

Number of observations 132197

Unique reflections 24354

Completeness (%)^b^ 98.4 (88.7)

I/σI^b^ 11 (1.8)

***Refinement***

Resolution (Å) 30 – 2.0

Number of reflections 20105

Final R^c^, R_FREE_ 0.209, 0.252

protein residues 285

solvent molecules 173

atoms^d^ 2546 (46)

Mean B-factor (Å^2^) 21

Rmsd bonds (Å) 0.007

Rmsd angles (˚) 1.3

Rmsd bonded Bs (Å^2^) 2.5/1.9

Number of TLS groups 1

Ramachandran (%) 92.3/6.9/0.4/0.4

^a^ Rsym = Σ||I| - |<I>||/Σ|<I>|, where I is the intensity of a single observation and <I> the average intensity for symmetry equivalent observations.

^b^ In parenthesis, for the highest resolution shell.

^c^ R = Σ|Fo-Fc|/Σ|Fo|, where Fo and Fc are observed and calculated structure factor amplitudes, respectively. R_FREE_ is calculated as R for reflections sequestered from refinement.

^d^ In parenthesis, the number of atoms assigned less than unit occupancy.
