# Supplementary material for: Mechanistic and Structural Understanding of Uncompetitive Inhibitors of Caspase-6
Source: PLoS One. 2012 Dec 5;7(12):e50864. doi: 10.1371/journal.pone.0050864 (PMC3515450; doi:10.1371/journal.pone.0050864)
Supplement: Experimental Procedures S1 — Supplemental Methods. (DOCX) [file pone.0050864.s008.docx]

SUPPLEMENTAL METHODS

*Expression and purification of caspase-6 for enzymatic assays:* Human caspase-6 was expressed as the mature enzyme using a split construct. Residues 24-179 and 194-293 from caspase-6 cDNA (Origene) were cloned into pET-24b and pET-23b vectors (Novagen) respectively; the small subunit contained a C-terminal 6x histidine tag. Vectors were cotransfected into the E.coli strain BL21 (DE3) pLysS (Stratagene). Cells were grown from a single colony in 6L of 2xYT supplemented with ampicillin and kanamycin at 37 ^o^C to an OD of ~ 0.5 and were subsequently cooled to 16 ^o^C before overnight induction with 0.2 mM IPTG. Cells were harvested by centrifugation, resuspended in 100 mM NaCl, 100 mM Tris, pH 8.0, and lysed. The supernatant was loaded onto a 1 mL HisTrap FF column (GE Healthcare), washed with 80% buffer A (100 mM NaCl, 100 mM Tris pH 8.0), 20% buffer B (100 mM NaCl, 100 mM Tris pH 8.0, 200 mM imidazole), and eluted with 100% B. The product was then diluted with 40 mL of 20 mM Tris pH 8, loaded onto a 1 mL HiTrap Q HP column (GE Healthcare), and eluted in a gradient of 0-50% 20 mM Tris pH 8.0, 1 M NaCl against 20 mM Tris pH 8.0 over 30 column volumes. Caspase-6 typically eluted with ~200 mM NaCl. Fractions were collected, concentrated by ultrafiltration and assayed for purity by denaturing gel electrophoresis and by mass spectrometry (LCT Premier; Waters). Expression of caspase-6 by this approach yielded 3-10 mg of pure protein per 6 liters of culture.

Active site titration was used to determine the concentration of active enzyme. 49 µL of 0.125 µM caspase-6 was incubated with increasing concentrations of the irreversible active site inhibitor, VAD-FMK (0-100 µM in DMSO), for four hours. The enzyme was then diluted 10-fold with assay buffer, and 25 µL were removed and added to 25 µL of 20 µM Ac-VEID-AFC to initiate enzymatic reaction. V/V_0_ was plotted as a function of inhibitor concentration; the intersection at the X-axis in the linear region of the curve denotes the concentration of active sites (100%).

*Cleavage of Lamin A by purified human Caspase-6***:** Recombinant caspase-6 was prepared at 6.6 nM final concentration in assay buffer (40 mM HEPES [pH 7.2], 100 mM NaCl, 1 mM EDTA, 2 mM DTT, 0.1% CHAPS, 3% DMSO), and pre-incubated at 37 °C with increasing concentrations (as indicated) of the compound to be assayed. After 30 minutes, GST-tagged Lamin A substrate (Abnova, Cat# H00004000-P01) was added to the reaction mix at a final concentration of 0.33 µM and the incubation was continued for 2 h at 37 °C. Samples were boiled for 5 min to terminate the proteolytic cleavage reaction before they were resolved by SDS-PAGE (10-20% Tris-glycine gel) and transferred to nitrocellulose. The blot was probed with mouse anti-Lamin A antibody (Abnova # H00004000-B01P) and detected with IRDye800 anti-mouse antibody (LI-COR #926-32210). The expected cleavage product had an apparent MW ~ 50 KDa. Imaging was done on the LI-COR Odyssey® Infrared Imaging System using the 800 nm channel.

*Molecular Modeling***:** The protein was prepared using the default settings in the Protein Preparation Workflow in Maestro (Maestro, version 9.1 Schrodinger, LLC, New York, NY, 2010).   An initial model of the tetrahedral intermediate formed by the addition of the catalytic cysteine to the backbone carbonyl carbon of the aspartate residue in the VEID-R110 substrate was obtained by using the covalent docking protocol contained in Prime (Prime, version 2.2, Schrodinger, LLC, New York, NY, 2010) with the caspase-6/**3** complex considered the receptor. As the protocol does not change the hybridization state, an sp^3^ model was used that would generate the correct adduct upon covalent attachment (below). The complete VEID-R110 substrate was then superimposed onto the ten covalently docked results generating forty results due to stereoisomerism and rotamers of the R110 moiety. All but one complex were discarded due to significant clashes with the protein. The side chains and loops in close proximity to the fluorophore in this complex (Glu121, Gly122, His123, Asn166, and Tyr217) were refined using Prime.  The resulting complex was then minimized in MacroModel using the OPLS_2005 forcefield. Ligand, substrate and Cys163 were allowed to move freely--all other atoms were constrained by fixing the force constant at 200. A Michaelis model was generated by using the covalent model as a starting point. The substrate was stripped from the complex, converted back to the sp^2^ ligand and minimized with MacroModel (MacroModel, version 9.8, Schrodinger, LLC, New York, NY, 2010). This substrate was placed back in the active site by superimposing the coordinates of all the atoms except for those attached to the carbonyl carbon that is attacked by the cysteine nucleophile. This complex was then minimized with ligand, substrate, and the catalytic dyad allowed to move freely and all other atoms constrained by fixing the force constant as described above.

**Chemical Syntheses:**

**5-(3,4-Dimethoxyphenyl)furan-3-carboxylic Acid (6**).

A microwave vial was charged with 3,4-dimethoxyphenylboronic acid (**5**) (200 mg, 1.1 mmol), 5-bromofuran-3-carboxylic acid (**4**) (210 mg, 1.1 mmol), tetrakis(triphenylphosphine)palladium(0) (130 mg, 0.11 mmol), and Na_2_CO_3_ (1 M aq., 2.2 mL) in 1,2-dimethoxyethane (2 mL). The vial was capped and purged with N_2_ for 10 min. The reaction mixture was heated in microwave reactor at 130 ^o^C for 20 min. After cooling to ambient temperature, the mixture was diluted with water (20 mL) and extracted with EtOAc (2 x 20 mL). The aqueous phase was acidified with citric acid (10% aq.). The precipitate was collected by filtration, washed with small amount of water, and air-dried. The solids were dissolved in CH_2_Cl_2_/EtOAc (1:1; 20 mL), filtered through a short pad of Celite, and concentrated to afford 210 mg (77%) of the title compound as a light brown solid: ^1^H NMR (400 MHz, D_6_-DMSO) δ 12.67 (s, 1 H), 8.28 (s, 1 H), 7.27 - 7.33 (m, 2 H), 7.13 (s, 1 H), 7.02 (d, *J* = 8.3 Hz, 1 H), 3.82 (s, 3 H), 3.78 (s, 3 H); LC-MS: *m/z* = 249 [M+H]^+^.

**Methyl (2*R*)-3-(3-Cyanophenyl)-2-[[5-(3,4-dimethoxyphenyl)furan-3-carbonyl]amino]propanoate (8)**. To a solution of acid **6** (50 mg, 0.20 mmol), EDAC (77 mg, 0.40 mmol), and HOBt (54 mg, 0.40 mmol) in CHCl_3_ (1.5 mL) were sequentially added (*R*)-methyl 2-amino-3-(3-cyanophenyl)propanoate hydrochloride (**7**) (73 mg, 0.30 mmol) and DIPEA (0.14 mL, 0.80 mmol). The resulting mixture was stirred at ambient temperature overnight then diluted with CH_2_Cl_2_ (10 mL), washed with HCl (0.5 N aq., 10 mL) and NaHCO_3_ (10% aq., 10 mL), dried over Na_2_SO_4_, filtered, and concentrated. The crude material was purified by preparative HPLC to afford 34 mg (43%) of the title compound as an off-white solid: ^1^H NMR (300 MHz, D_6_-DMSO) δ 8.64 (d, *J* = 8.1 Hz, 1 H), 8.19 (s, 1 H), 7.78 (s, 1 H), 7.69 (d, *J* = 7.6 Hz, 1 H), 7.64 (d, *J* = 7.9 Hz, 1 H), 7.51 (t, *J* = 7.7 Hz, 1 H), 7.25 (s, 2 H), 7.13 (s, 1 H), 7.04 (d, *J* = 9.0 Hz, 1 H), 4.75 (ddd, *J* = 9.8, 9.8, 5.4 Hz, 1 H), 3.83 (s, 3 H), 3.80 (s, 3 H), 3.66 (s, 3 H), 3.25 (dd, *J* = 13.8, 5.4 Hz, 1 H), 3.10 (dd, *J* = 13.7, 10.1 Hz, 1 H); ^13^C NMR (75 MHz, D_6_-DMSO) δ 171.7, 161.6, 154.0, 149.0, 148.9, 144.1, 139.2, 134.2, 132.8, 130.4, 129.4, 123.7, 122.5, 118.8, 116.3, 112.1, 111.1, 107.4, 103.1, 55.5, 55.5, 53.0, 52.0, 35.7; HRMS calcd for C_24_H_23_N_2_O_6_ [M + H]^+^ 435.1556, found 435.1996.

***N*-[(1*R*)-1-[(3-Cyanophenyl)methyl]-2-hydroxy-ethyl]-5-(3,4-dimethoxyphenyl)furan-3-carboxamide (3).** To a solution of ester **8** (65 mg, 0.15 mmol) in THF (1.0 mL) was added NaBH_4_ (11 mg, 0.30 mmol). The reaction was stirred at ambient temperature for 2.5 h. Additional NaBH_4_ (11 mg, 0.30 mmol) was added, and the mixture stirred overnight. More NaBH_4_ (11 mg) was added. After 4 h, the reaction was quenched by dropwise addition of water. The crude product was extracted with CH_2_Cl_2_ (2 x 10 mL). The combined organics were dried over Na_2_SO_4_, filtered, and concentrated. Purification by preparative HPLC afforded 49 mg (81%) of the title compound as an off-white solid: ^1^H NMR (300 MHz, D_6_-DMSO) δ 8.16 (s, 1 H), 7.96 (d, *J* = 8.6 Hz, 1 H), 7.71 (s, 1 H), 7.57 - 7.69 (m, 2 H), 7.48 (t, *J* = 7.7 Hz, 1 H), 7.20 - 7.30 (m, 2 H), 7.14 (s, 1 H), 7.03 (d, *J* = 8.9 Hz, 1 H), 4.93 (t, *J* = 5.5 Hz, 1 H), 4.16 (m, 1 H), 3.83 (s, 3 H), 3.80 (s, 3 H), 3.39 - 3.56 (m, 2 H), 3.03 (dd, *J* = 13.7, 4.7 Hz, 1 H), 2.81 (dd, *J* = 13.7, 9.4 Hz, 1 H); ^13^C NMR (75 MHz, D_6_-DMSO) δ 161.2, 153.7, 149.0, 148.8, 143.7, 141.0, 134.1, 132.6, 129.8, 129.3, 124.5, 122.6, 118.9, 116.2, 112.1, 111.0, 107.3, 103.2, 62.9, 55.5, 55.5, 52.1, 36.0; HRMS calcd for C_23_H_23_N_2_O_5_ [M + H]^+^ 407.1607, found 407.2056.
